# MmPalateMiRNA, an R package compendium illustrating analysis of miRNA microarray data

**DOI:** 10.1186/1751-0473-8-1

**Published:** 2013-01-08

**Authors:** Guy N Brock, Partha Mukhopadhyay, Vasyl Pihur, Cynthia Webb, Robert M Greene, M Michele Pisano

**Affiliations:** 1 Department of Bioinformatics and Biostatistics, University of Louisville, Louisville, KY, USA; 2 Birth Defects Center, University of Louisville, Louisville, KY, USA; 3 Department of Molecular Cellular and Craniofacial Biology, University of Louisville, Louisville, KY, USA

## Abstract

**Background:**

MicroRNAs (miRNAs) constitute the largest family of noncoding RNAs involved in gene silencing and represent critical regulators of cell and tissue differentiation. Microarray expression profiling of miRNAs is an effective means of acquiring genome-level information of miRNA activation and inhibition, as well as the potential regulatory role that these genes play within a biological system. As with mRNA expression profiling arrays, miRNA microarrays come in a variety of platforms from numerous manufacturers, and there are a multitude of techniques available for reducing and analyzing these data.

**Results:**

In this paper, we present an analysis of a typical two-color miRNA microarray experiment using publicly available packages from R and Bioconductor, the open-source software project for the analysis of genomic data. Covered topics include visualization, normalization, quality checking, differential expression, cluster analysis, miRNA target identification, and gene set enrichment analysis. Many of these tools carry-over from the analysis of mRNA microarrays, but with some notable differences that require special attention. The paper is presented as a “compendium” which, along with the accompanying R package MmPalateMiRNA, contains all of the experimental data and source code to reproduce the analyses contained in the paper.

**Conclusions:**

The compendium presented in this paper will provide investigators with an access point for applying the methods available in R and Bioconductor for analysis of their own miRNA array data.

## Background

Much of the recent bioinformatics literature has focused on the role small RNA molecules, termed microRNAs (miRNAs), play in regulating gene expression within plant and animal systems [1]. Mature miRNAs are typically 18-25 bases in length and have been found to execute key functions in silencing expression of specific target genes [2]. MicroRNAs regulate expression of genes post-transcriptionally, by binding the target mRNA molecule and either directly inhibiting translation or destabilizing the target mRNA [3]. MicroRNA microarray technology has been successfully exploited to generate microRNA gene expression profiles of the cell cycle [4], cell differentiation [5], cell death [6], embryonic development [7], stem cell differentiation [8], different types of cancers [9,10], the diseased heart [11] and diseased neural tissue [12]. Thus, microRNA gene expression profiling offers an effective means of acquiring novel and valuable information regarding the expression and regulation of genes, under the control of miRNAs, in a variety of biological systems.

The R software programming language [13] has gained wide popularity among the scientific research community, along with its extension to the realm of genomics applications via the Bioconductor [14,15] software for bioinformatics project. The Bioconductor project contains a variety of R packages for application to high-throughput “omics” data, including array preprocessing and normalization, identification of differentially expressed genes, clustering, classification, gene-set enrichment analysis, and other down-stream analysis methods. Hence, the R packages available at Bioconductor can provide a complete suite of tools for analyzing array data from the initial preprocessing steps through the final determination of interesting genes and gene sets. Several publications have addressed how to perform and reproduce an analysis of mRNA expression array data using software from R and Bioconductor [16-18]. An integrated way to present the analysis from these experiments is in the form of a compendium [17,18], which encapsulates the primary data, supporting software, statistical analysis, and document text in a manner that allows other investigators to completely reproduce the results of the experiment.

While many of the same tools for analyzing mRNA expression arrays can be applied to the analysis of miRNA data, there are distinct differences between the two platforms which necessitate special use of some methods (see overviews by Sarver [19] and Thomson *et al.*[20]). In particular, miRNA arrays typically have far fewer genes that are spotted on the array compared to mRNA arrays and require careful consideration of the assumptions behind array pre-processing methods prior to their application. Several recent publications have compared various normalization methods for microRNA microarray data [21-23], while others have developed novel methods specifically for miRNA data [24-27]. Though certain methods were found to outperform others in each case, in general there is still no consensus on the best normalization method. Therefore, investigators are encouraged to perform their own assessments to determine an appropriate normalization method for their data. A second unique aspect of miRNA analysis relative to mRNA analysis is that differentially expressed miRNAs are subsequently evaluated for potential gene targets that are regulated by the miRNAs. A number of databases can be used for this purpopse, and many of these have been ported to R in the form of Bioconductor packages. It is these putative regulatory targets that are typically evaluated for biological and molecular functionality, e.g. by gene set enrichment analysis.

In this article, we illustrate how to analyze a two-color miRNA experiment using available packages from Bioconductor and the Comprehensive R Archive Network (CRAN). Example code is provided for the complete analysis including preprocessing of arrays, normalization, identification of differentially expressed miRNAs, clustering, miRNA target identification, and gene set enrichment analysis. The analysis presented here follows closely to what was presented by Mukhopadhyay *et al.*[28]. Aspects of miRNA analysis which require special attention are highlighted, as are particular advantages of using specific R and Bioconductor packages. Although the analysis is specific to the Miltenyi Biotech miRXplore platform [29], the general steps outlined here can easily be extended to other platforms as well. To ensure reproducibility of the results, the entire analysis is presented as a compendium [17,18], in the form of an accompanying R package called MmPalateMiRNA [30], which has been made freely available on Bioconductor. The package also includes several functions to produce diagnostic plots for evaluating probe intensity distributions on miRNA microarrays, as discussed in Sarkar *et al.*[26]. The experimental data used in this manuscript are freely available as part of the compendium package (GEO DataSets [31], accession number GPL10179).

## Methods

In the following subsections, we discuss the methodologies used for the analysis of the miRNA data in this compendium. We refer the reader to the original papers for detailed methods, here just providing an overview.

### Preprocessing

An important first step in the analysis of microarray data is to check the array quality by inspecting for outliers, spatial artifacts, and for differences in array intensity distributions which may require normalization. Several software packages exist for this purpose; in particular, the arrayQualityMetrics package [32] available from Bioconductor provides a comprehensive report for both one and two-color microarray data. However, the diagnostic plots in that package for two-color arrays are constructed from ratios of the two channels (M values), and for miRNA data plots focused solely on the control / reference channel may be more relevant. Specifically, Sarkar *et al.*[26] introduced novel diagnostic plots for miRNA data for the purpose of evaluating and comparing different normalization methods, which serve as useful indicators for array quality and outlyling arrays. In addition to evaluating array quality, other important pre-processing steps include identifying outlying values for specific probes, performing non-specific filtering of probes, and imputing probes that are missing or are extreme outliers.

### Normalization

Several recent publications have drawn attention to the normalization of miRNA data as distinct from that of mRNA data. In particular, methods that assume some level of symmetry in differential expression, such as loess and quantile normalization, may be inappropriate when global changes associated with phenotypes are present [20]. As such, normalization methods that use a set of invariant probes [23,26], or use single-channel normalization methods [21] may outperform so-called “global” normalization methods. Recent comparisons of normalization methods for miRNA microarray data have resulted in differing conclusions [21-23], with top performing methods ranging from quantile normalization for single-channel array data [22] to print-tip loess for two-channel data [21]. However, Sarkar *et al.*[26] evaluated several different normalization methods, incluuding variance stabilizing normalization (VSN) [33], spike-in VSN, and print-tip loess, and found no statistically significant differences between them based on correlation with qRT-PCR measurements. As is typical with array data in general, investigators are encouraged to try several different normalization methods and evaluate the differences betweeen them on the basis of diagnostic plots [26].

### Differential expression

A variety of methods exist to determine differential expression between two or more groups of expression data, including the classic *t*-test and the more recent ‘moderated’ variants. Members of the latter category include the Significant Analysis of Microarrays (SAM) [34], and empirical Bayes methods [35,36]. In particular, the methodology developed by Smyth [36] extends these concepts to apply to general microarray experiments with arbitrary numbers of treatments and samples, in the context of a hierarchical linear model. A model is fitted to the expression values for each gene/transcript, and used to evaluate differential expression for contrasts (comparisons between treatment groups) of interest. A ‘shrinkage’ estimate of the variability is obtained by a weighted average of the a pooled estimate of variation and the per-gene estimate of variation. This lessens the occurrence of large *t*-statistics due to exceptionally small variance estimates, and effectively introduces a “fold-change” criterion into the statistic. The methods are available in the Bioconductor package limma [37].

### Clustering

Clustering of array profiles is helpful for determining underlying structure in the changes of gene expression, especially for time course data. Common methods include hierarichical clustering, divisive hierarchical clustering (DIANA), K-means, self-organizing maps (SOM), the self-organizing tree algorithm (SOTA), partitioning around medoids (PAM), and model-based clustering [38-40]. With the diversity of methods available for the investigator to try, a commonly encountered difficulty is determining which clustering algorithm to use for a particular data set. This problem can be partially overcome using clustering validation measures, as found in the clValid package [41]. The clValid package allows the user to select from among ten different clustering algorithms and uses three different sets of validation measures (internal, stability, and biological) to evaluate the performance of each algorithm for a range of cluster numbers.

### Identification of miRNA target genes

After a subset of miRNAs of interest has been determined, e.g by differential expression or clustering, the next step is to determine the potential regulatory targets of the miRNA molecules. Algorithms for predicting miRNA target molecules are fundamentally based on sequence complimentarity (between the mature miRNA transcript and the 3’-untranslated regions of potential target mRNAs), species conservation, thermodynamic stability, and site accessibility (see Alex *et al.*[42] for an overview). The Bioconductor package RmiR.Hs.miRNA [43] contains six databases for human miRNA targets, while the database of targets in miRBase [44] is available through the Bioconductor packages mirbase.db [45] and microRNA [46]. The TargetScan database of miRNA targets [47] is also available in targetscan.Hs.eg.db [48] for humans and targetscan.Mm.eg.db [49] for mouse.

### Gene set analysis

Once putative regulatory targets of the differentially expressed miRNAs have been identified, a logical next step is to identify what biological or functional pathways the targets have in common with each other. This can be accomplished by gene set analysis, or gene set enrichment analysis [50]. The regulatory targets are compared with predefined gene sets such as GO classifications [51], KEGG pathways [52], chromosome bands, and protein complexes. Gene set analysis is based on the hypergeometric test and identifies which biological gene sets have an under- and over-representation of the identified miRNA targets. Bioconductor packages which provide gene set analysis include GOstats [53] and Category [54].

## Results and discussion

### Preliminaries

R packages that are needed for running the example code in this manuscript are MmPalateMiRNA [30] and its dependencies, and the additional packages latticeExtra [55], clValid [41], targetscan.Mm.eg.db [49], microRNA [46], org.Mm.eg.db [56], and GOstats [53]. The full list of dependencies is given in the **Availability and requirements**. To begin, we download and install all of the needed packages for running the code in this compendium. In the following, text after the R> prompt denotes an R command, and a “+” denotes a continuation in code. The R code from this compendium is available as Additional file 1 (“MmPalateMiRNA_SCBM.R”).

Next, we load the MmPalateMiRNA package, which additionally loads the required packages Biobase [14], limma [37], vsn [33], statmod [57], lattice [58], and xtable [59]. Further, we load the remaining needed packages for running the code in this compendium.

### miRNA data

The microRNA microarray data in this compendium were obtained as previously described in Mukhopadhyay *et al.*[28], and the data are publicly available from GEO [31] (accession number GPL10179). Briefly, mouse embryonic tissue was obtained on gestational days (GD) 12, 13, and 14, which represents the critical period of palate development in the mouse. Total RNA (containing miRNAs) was isolated using standard RNA extraction protocols. RNA samples (1 *μ*g) isolated from mouse embryonic orofacial tissues (GD-12 - GD-14) as well as the miRXplore Universal Reference (UR, control channel) were fluorescently labeled with Hy5 (red) or Hy3 (green), respectively, and hybridized to Miltenyi Biotec miRXplore Microarrays using the a-Hyb Hybridization Station [29]. For each gestational day, three distinct pools of RNA were independently processed and applied to microarray chips. Probes for a total of 1336 mature miRNAs (from human, mouse, rat and virus), including positive control and calibration probes, were spotted in quadruplicate on each microarray. Each array included probes for 588 murine miRNAs. The miRXplore Universal Reference (UR) controls, provided by Miltenyi, represent a defined pool of synthetic miRNAs for comparison of multiple samples. Fluorescence signals of the hybridized miRXplore Microarrays were detected using a laser scanner from Agilent Technologies. Mean and median signal and local background intensities for the Hy3 and Hy5 channels were obtained for each probe on each of the nine microarray images using the ImaGene software [60]. The experimental data is included in the MmPalateMiRNA package in a compiled format, as an RGList object (a class in package limma [37] for two-color microarray data) called PalateData. The data is loaded into the R session using the code below. To see how PalateData was created from the source data files, see Additional file 2 (“ReadingTwoColorData.pdf”) and the corresponding R code in Additional file 3 (“ReadingTwoColorData.R”). For more information on the data in PalateData, use ?PalateData or see Additional file 2.

### Preprocessing

#### Outlying arrays

**Figure 1:**
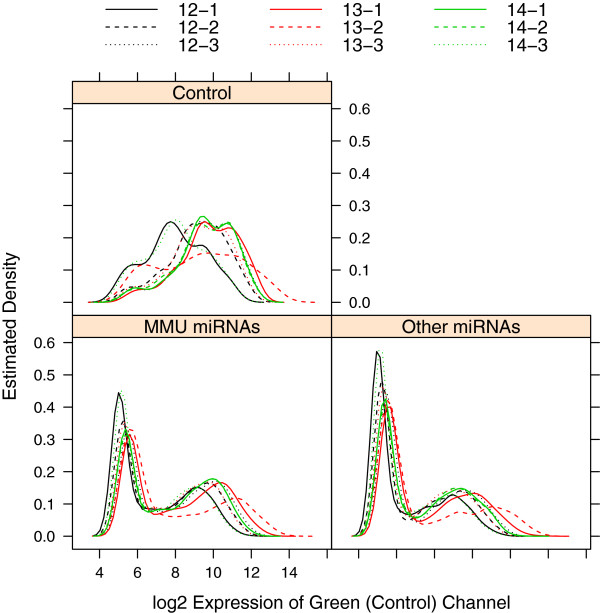
**Estimated density of reference channel before normalization.** Estimated density of the log2 intensity values of the reference (control) channel in the PalateData miRNA data from the MmPalateMiRNA package. Separate panels are provided for “MMU miRNAs”, “Other miRNAs”, and “Control” probes. Lines are color-coded according to gestational day (GD 12 = black, GD 13 = red, GD 14 = green), and different line types represent replicates within each GD.

Sarkar *et al.*[26] described several diagnostic plots for miRNA data that can be used to evaluate the need and effectiveness of normalization procedures. These plots can also serve as aids to determine outlying arrays and batch effects. One such plot is the kernel density estimate for each array, for different types of probes. Figure 1 plots the density estimates of the log2 intensity values in the control channel for the unnormalized data, separated into “MMU miRNAs” (MMU = Mus musculus, i.e. mouse), “Other miRNAs”, and “Control” probes (other probes were non-informative). The plot requires use of the lattice package, and the MmPalateMiRNA package contains methods to produce plots for RGList objects based on the generic functions in lattice. The code below illustrates the use of the function densityplot to produce Figure 1. To access the documentation file for this function, use ?densityplot (in general, the documentation file for function fun is accessed through ?fun, and the documentation file for S4 class obj is accessed through class?obj).

**Figure 2:**
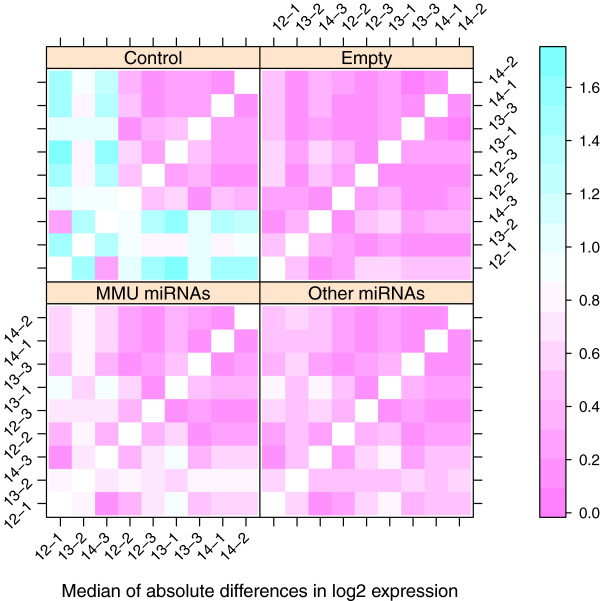
**Distance between arrays prior to normalization.** Distance between arrays in the PalateData miRNA data from the MmPalateMiRNA package. Distance was based on the median of the absolute differences in unnormalized log2 intensity values of the reference channel. Separate panels are provided for “MMU miRNAs”, “Other miRNAs”, “Control”, and “Empty” probes. Arrays have been reordered so that the outlying arrays (12-1, 13-2, and 14-3) are grouped together.

Figure 1 indicates three possible outlying arrays, GD 12-1, 13-2, and 14-3. A second figure (Figure 2) can be constructed based on the pairwise “distance” between arrays, as measured by the median of the absolute differences in log2 intensity values for miRNAs in the green channel [26]. The plot is created using the levelplot method for RGList objects, which is included in the package. Here we separate the plots according to the type of probe, and the arrays are reordered so that the outlying arrays are grouped together. The three arrays are clearly outliers based on the control probes, but to a lesser extent based on the other types of probes.

Figures 1 and 2 demonstrate the potential need for normalization or removal of several of the arrays. In the **Normalization** subsection, we will evaluate the effectiveness of several normalization methods in correcting these systematic differences between the arrays.

#### Outlying values

In addition to checking for outlying arrays, it is important to check for outlying values on individual probes. To accomplish this, we evaluated for each probe whether there were any extreme values (greater than 2.665 standard deviations above the mean). The checkOutliers function checks this for each of the red and green channels in an RGList object and returns the indices of array probes with extreme values.

The probes with outlying arrays can be visualized using boxplots with the code below.

The figure is omitted but clearly shows that the identified outlying values are nearly two orders of magnitude above the rest of the intensity values. Rather than omitting these values, we exploit the replicated design of the arrays and substitute the mean of the other replicates on the array for the extreme values using the fixOutliers function.

#### Missing values

In addition to checking for outlying values, we also check for any missing values in the two channels using the checkMVs function. Here, we only find two probes on the array with missing values in the background channels, so we again impute these values using the means of the backgrounds from the other three replicates on the chip using the fixMVs function.

#### Filtering probes

Prior to running the normalization methods, we filter the probes and keep only those which correspond to miRNAs and calibration probes. Additionally, probes that are not sufficiently above the background intensity level may be unreliable and represent noise that can interfere with subsequent analysis, including normalization [26]. Prefiltering also reduces the number of statistical comparisons being performed and improves overall power [61]. Here, we filter probes whose foreground intensity values are below 1.1 times their background intensity level. To allow for probes which may be expressed for a particular experimental condition (here, gestational day), we keep all probes which have at least 3 samples above the filtering threshold. Lastly, only those genes with all four replicates passing the filtering step are retained. After all pre-processing steps, a total of 956 probes, corresponding to 175 mouse miRNAs, 42 other miRNAs, and 22 calibration probes each replicated 4 times, remain.

### Normalization

Based on the literature [21-23,26], we evaluated several normalization procedures on the filtered data, including none, median, loess, quantile, VSN, and spike-in VSN. The limma package [62] includes various options for both within (normalizeWithinArrays) and between (normalizeBetweenArrays) array normalization, and the vsn package [33] has functions for performing VSN and spike-in VSN. In all cases, a simple background correction was performed by subtracting background from the foreground intensities.

#### Diagnostic plots

**Figure 3:**
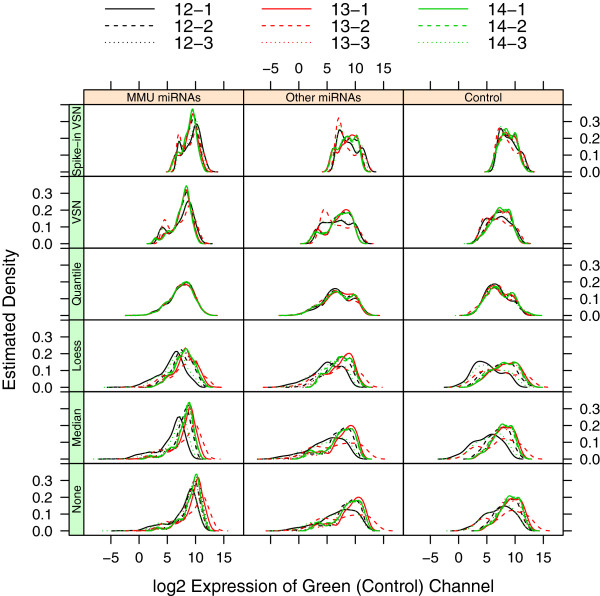
**Density of reference channel after normalization.** Estimated density of the log2 intensity values of the reference channel in the PalateData miRNA data from the MmPalateMiRNA package, both before (“None”) and after normalization by various normalization procedures. Separate panels are provided for “MMU miRNAs”, “Other miRNAs”, and “Control” probes. Lines are color-coded according to gestational day (GD 12 = black, GD 13 = red, GD 14 = green), and different line types represent replicates within each GD.

Several diagnostic plots can be used to contrast the effectiveness of each normalization procedure. The MmPalateMiRNA package contains several methods to produce these plots for lists of class MAList or NChannelSet objects, based on functions in the lattice package. Figure 3, rows one through five, plots the intensity distribution for the reference channels after each of the normalization procedures (use of the useOuterStrips function requires the latticeExtra package). Note that the order of panels in lattice plots is from the bottom left panel to the right and up, according to the rules used for graphs. The quantile normalization procedure is clearly the most successful in removing the intensity bias that was apparent for three of the arrays (12-1, 13-2, and 14-3), while loess and median normalization appear to be the least successful. Notably, normalization based on the spike-in probes was unsuccessful, perhaps since these probes were shifted differently in the reference channel relative to the other probe types.

**Figure 4:**
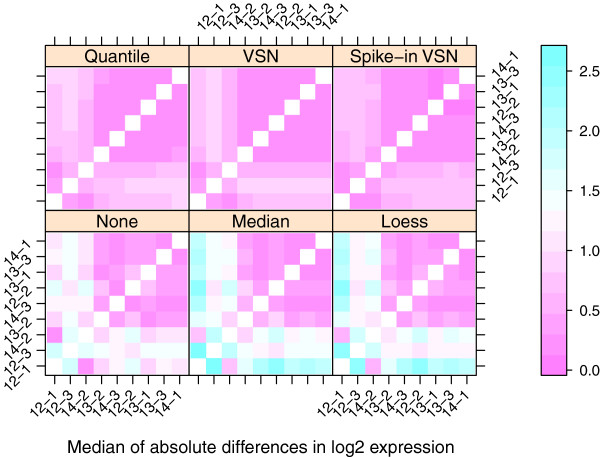
**Distance between arrays in reference channel after normalization.** Distance between arrays in the PalateData miRNA data from the MmPalateMiRNA package, both before (“None”) and after normalization by various normalization procedures. Distance was based on the median of the absolute differences in log2 intensity values of the reference channel, for all probes remaining after filtering. Arrays have been reordered so that the outlying arrays (12-1, 13-2, and 14-3) are grouped together.

An additional plot based on the median absolute difference between probes in the reference channel can be used to compare relative success of the normalization procedures in removing the array effect (Figure 4). Here again, quantile normalization appears to be the best, while loess and median normalization are the least effective.

**Figure 5:**
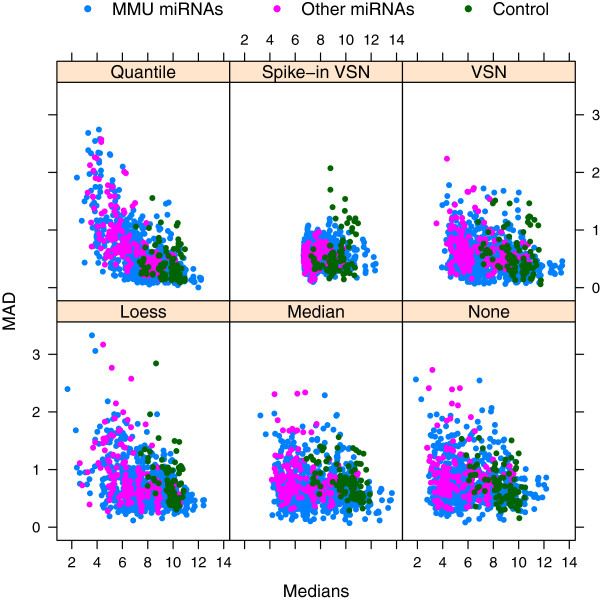
**Spread versus location of probes for the experimental channel.** Spread, as measured by the median absolute deviation (MAD), versus median expression for each probe remaining after filtering in the experimental channel of the PalateData miRNA data from the MmPalateMiRNA package. Separate panels are provided for data both before (“None”) and after normalization by various normalization procedures. Points are color-coded by type of probe (“MMU miRNAs”, “Other miRNAs”, and “Control”).

To investigate the effect of the normalization procedure on the experimental channel, plots of the spread (median absolute deviation) versus the location (median) of all probes can be used. Plots of this type can be produced using the MADvsMedianPlot function in the MmPalateMiRNA package. Probes of different types are highlighted, with particular focus on the spike-in probes, which should have low variability across all the arrays. In Figure 5, spike-in VSN has the lowest variability among the spike-in probes, compared to the other normalization methods. However, spike-in VSN has also dramatically decreased the variation among *all* the probes in the experimental channel, making the normalization procedure questionable in this case. Quantile normalization has resulted in large variations for some of the probes with lower intensity values.

**Figure 6:**
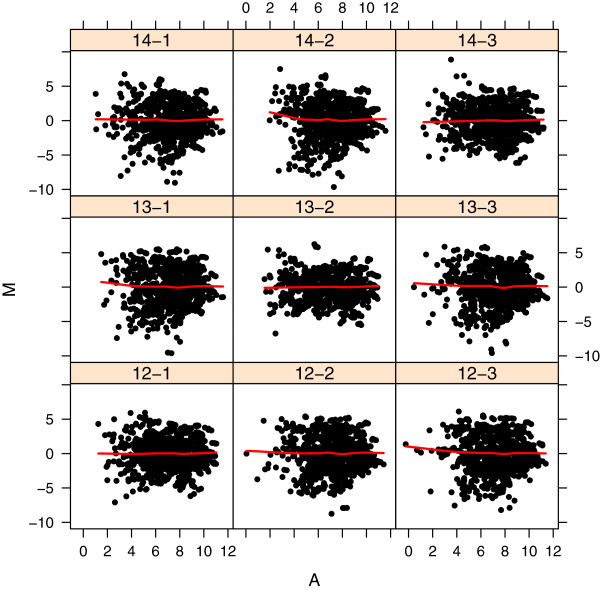
**Log intenstiy ratios (M) versus average intensity values (A) after quantile normalization.** Log_2_ intensity ratios (M values) plotted against average log2 intensity values (A values) for each probe, for each array of the PalateData miRNA data from the MmPalateMiRNA package after quantile normalization. Red lines are loess smoothed regression lines for each M versus A comparison.

Plots of the log2 intensity ratios (M values) versus the mean log2 intensity values (A values) for each probe can be used to evaluate whether there is a bias associated with overall intenstity level for each array. This so-called “MA” plot is illustrated in Figure 6 for quantile normalization. MA plots for the other normalization methods are not shown, though code to produce the plots is available in the accompanying R script “MmPalateMiRNA_SCBM.R”. Quantile normalization has removed any association between the M and A values, while for VSN normalization there is still a trend which is similar to the unnormalized data. The MA plot for spike-in VSN shows a dramatic effect on the intensity ratios.

**Figure 7:**
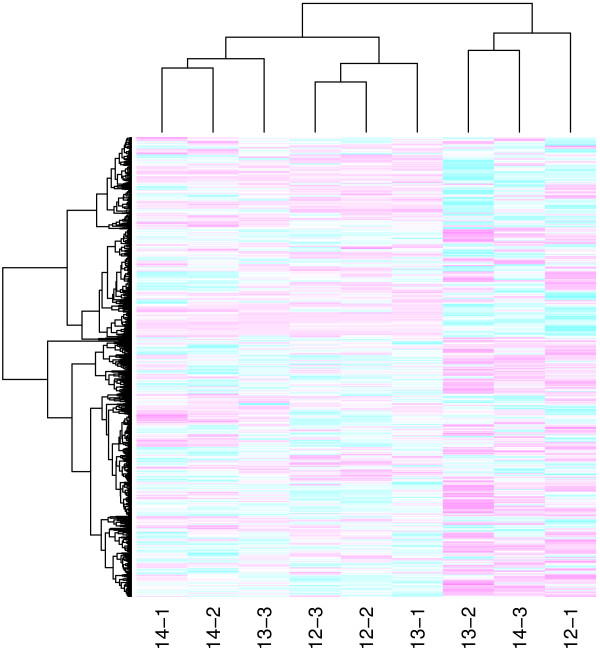
**Heatmap of *log2 *intensity ratios after quantile normalization.** Heatmap of log2 intensity ratios (expression values) of the PalateData miRNA data after quantile normalization. Arrays and probes are clustered by hierarchical clustering. Arrays 12-1, 13-2, and 14-3 do not cluster with the other replicates for the corresponding gestational day.

As a final evaluation, we inspected heatmaps along with hierarchical clustering of the arrays. Figure 7 displays the heatmap after quantile normalization and reveals that the previously identified outlying arrays (samples 12-1, 13-2, and 14-3) still do not cluster with the other replicates for that day.

Table 1 gives the correlations between each pair of arrays, based on the log2 intensity ratios. Since the other two replicates for each day were highly correlated (*r*≥0.95), we decided to use only those two replicates from each day for subsequent statistical analysis. Normalization was redone omitting the arrays 12-1, 13-2, and 14-3, using quantile normalization.

**Table 1:** Correlation between arrays after quantile normalization

	**12-1**	**12-2**	**12-3**	**13-1**	**13-2**	**13-3**	**14-1**	**14-2**	**14-3**
12-1	1.00	0.86	0.86	0.85	0.86	0.84	0.82	0.83	0.88
12-2	0.86	1.00	0.97	0.96	0.86	0.96	0.92	0.93	0.89
12-3	0.86	0.97	1.00	0.96	0.85	0.94	0.91	0.93	0.87
13-1	0.85	0.96	0.96	1.00	0.86	0.95	0.94	0.94	0.90
13-2	0.86	0.86	0.85	0.86	1.00	0.85	0.83	0.84	0.90
13-3	0.84	0.96	0.94	0.95	0.85	1.00	0.95	0.95	0.87
14-1	0.82	0.92	0.91	0.94	0.83	0.95	1.00	0.96	0.90
14-2	0.83	0.93	0.93	0.94	0.84	0.95	0.96	1.00	0.88
14-3	0.88	0.89	0.87	0.90	0.90	0.87	0.90	0.88	1.00

#### Imputation

Sixteen probes from the six arrays exhibited negative intensities after the background procedure and resulted in missing values for subsequent calculation of the log2 intensity ratios. A significant percentage of missing values can have a substantial impact on downstream analysis of array data [63], and in such cases choice of a imputation procedure should be carefully considered. Here, with a relatlively small percentage of missing values, the impact on data analysis will be relatively minimal. Hence we use the K-nearest neighbor imputation scheme [64] as a fast and effective approach, implemented in the imputeKNN function included in package MmPalateMiRNA.

### Determining differentially expressed miRNAs

To test for differential expression of miRNAs between different gestational days (GD-12, 13, and 14), the limma package [36,37] was used. Use of the limma package requires the user to create a design matrix, which defines the possible levels for each experimental factor, and is used to construct a model matrix and contrasts to test for differential expression between factor levels. The model matrix consists of indicator variables for the levels of each experimental factor in our design, which in our case corresponds to each of the gestational days.

Estimates of gene expression are based on the log2 Red/Green intensity ratios, hereafter referred to as ‘expression values’. Contrasts defined here estimate the differences in mean expression between each gestational day. The makeContrasts function in limma will generate these for you.

Some advantages of using limma over other methods include the ability to incorporate probe quality weights and to handle duplicate probes for each miRNA on the chip via the duplicateCorrelation function [62]. These advantages are particularly evident in small sample sizes, as in this experiment. To make use of the duplicated probes, we first order the normalized data so that replicated probes are adjacent to each other. The probe quality weights are incorporated in the calculation of the correlation matrix for the duplicated probes.

Next, the lmFit function is used to fit the hierarchical linear model, and the contrasts.fit function used to get contrast estimates. The eBayes function generates the moderated (empirical Bayesian) *t*-statistics corresponding to each of the contrast estimates.

The topTable function calculates and reports fold change, moderated *t*-statistics, unadjusted and adjusted *p*-values for the comparison of interest. *P*-values are adjusted by the method of Benjamini & Hochberg [65], which controls the expected false discovery rate. Code below shows the calculation for the comparison between gestational days 13 and 12, and the results are given in Table 2. Results for comparisons between the other gestational days are omitted but code to calculate them is included in the R script “MmPalateMiRNA_SCBM.R”.

**Table 2:** Significantly differentially expressed miRNAs for GD 13 versus 12

**miRNA Name**	**Organism**	**Fold Change**	**T-stat**	**Adj p-value**
LET-7B	MMU miRNAs	1.78	7.72	¡ 0.001
MIR-193A-3P	MMU miRNAs	2.94	6.85	¡ 0.001
LET-7C	MMU miRNAs	1.50	5.74	0.001
MIR-140-5P	MMU miRNAs	1.46	5.31	0.001
MIR-342	Other miRNAs	0.56	-5.18	0.001
MIR-31	MMU miRNAs	1.56	4.98	0.002
MIR-193B	MMU miRNAs	1.66	4.86	0.002
MIR-301	Other miRNAs	0.78	-4.44	0.005
MIR-20B	Other miRNAs	0.75	-4.37	0.006
MIR-543-3P	MMU miRNAs	0.69	-3.91	0.015
MIR-301B	Other miRNAs	0.71	-3.83	0.015
MIR-342-3P	MMU miRNAs	0.58	-3.83	0.015
MIR-22	MMU miRNAs	1.34	3.78	0.015
LET-7I	MMU miRNAs	1.35	3.75	0.015
MIR-152	MMU miRNAs	1.25	3.75	0.015
MIR-298	MMU miRNAs	0.77	-3.45	0.030
MIR-148A	MMU miRNAs	1.34	3.41	0.030
MIR-210	MMU miRNAs	1.33	3.40	0.030
MIR-422A	Other miRNAs	1.67	3.34	0.033
MIR-23A	MMU miRNAs	1.29	3.32	0.033
MIR-20A	MMU miRNAs	0.79	-3.30	0.033
MIR-347	Other miRNAs	1.19	3.18	0.042

Table 2: miRNA name, organism, fold change, moderated *t*-statistic, and adjusted *p*-values for comparisons in miRNA expression between gestational days 13 and 12. miRNAs which are up-regulated on GD 13 are indicated by fold-changes above one. *P*-values are adjusted by the method of Benjamini & Hochberg [65], which controls the expected false discovery rate.

**Figure 8:**
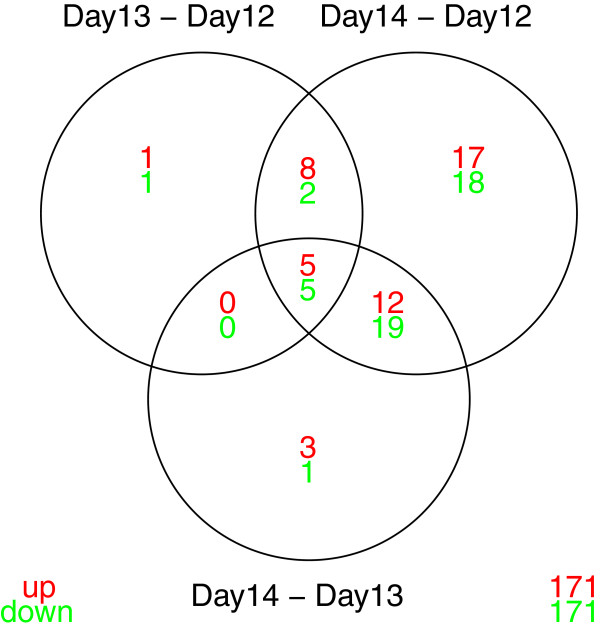
**Venn diagram for comparisons between gestational days.** Venn diagram illustrating the number of up- and down-regulated miRNAs for each comparison between gestational days, along with the number in the intersection of these sets. Includes all miRNAs from the PalateData miRNA data with an adjusted *p*-value ¡ 0.05 for at least one of the comparisons.

A nice summary of the results for the comparisons between gestational days is a Venn diagram, which gives the number of up- and down-regulated genes for each comparison, along with the number in the intersection of these sets (Figure 8).

Although we have focused on the calculation of test statistics corresponding to pairwise comparisons between gestational days, it is easy to obtain estimates for other contrasts of interests between the experimental conditions. For example, the the contr.poly function will provide contrasts to test for linear and quadratic trends, and the contr.helmert function gives the Helmert contrasts. To illustrate, we calculate analysis of variance (ANOVA) *F*-statistics for testing for differential expression between all three gestational days by combining two orthogonal contrasts, here using the contr.helmert function.

Next, the miRNAs with significant *F*-statistics (adjusted *p*<0.05) are identified for follow up examination, e.g. by clustering. The duplicates are averaged prior to further analysis.

### Clustering expression profiles

After identifying the differentially expressed miRNAs, clustering analysis can be performed to group genes with similar trends over time. A common difficultly is deciding which clustering algorithm to use and how many clusters to create. Cluster validation measures, as contained in the R package clValid [41], can help in this regard. Below, the clValid function is used to evaluate hierarchical clustering, SOTA, DIANA, and K-means clustering algorithms, for a range of one to six clusters in each case. The expression values for each day are averaged over the two replicates prior to clustering (object aveExpr). The internal validation measures (connectivity, Dunn Index, and Silhouette Width) are used with a correlation metric. A summary of the result indicates that hierarchical clustering with six clusters provides the optimal connectivity and Dunn Index measures, while DIANA with six clusters gives the optimal Silhouette Width.

**Figure 9:**
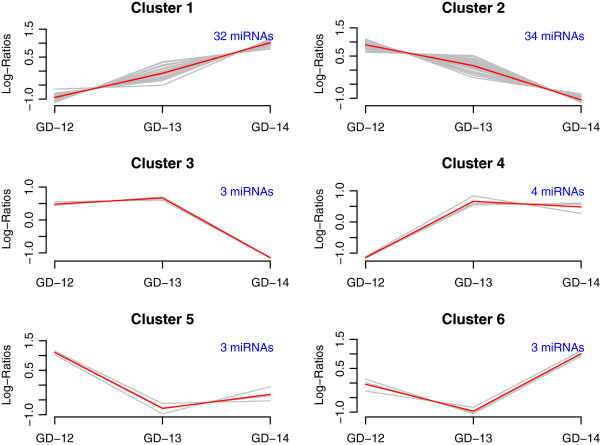
**Clustering of differentially expressed miRNAs.** Plot of clustering results for all significant (adjusted *p*-value for *F*-statistic ¡ 0.05) differentially expressed miRNAs from the PalateData miRNA data. Profiles are based on hierarchical clustering with six clusters, with expression values for each miRNA scaled to mean zero and standard deviation one.

The results from hierarchical clustering with six clusters was subsequently selected for visually displaying the data, using the clustPlot function available in package MmPalateMiRNA. The expression values for each miRNA are scaled to mean zero and standard deviation one for ease of visualization. The display is given in Figure 9. The two predominant clusters are cluster one and cluster two, which correspond to those miRNAs which exhibit a linear upward and downward trend over the time course, respectively.

### Determining miRNA target genes

To follow-up the results from the differentially expression and clustering analysis, the next step is to determine putative regulatory targets of the differentially expressed miRNAs. To illustrate, we identify the putative targets of the miRNAs contained in the first cluster in Figure 9. The miRNAs in the first cluster are evaluated for putative targets using the databases TargetScan [47] (package targetscan.Mm.eg.db [49]) and miRBase [44] (package microRNA [46]). The mouse specific miRNA names are first extracted and then converted to the standard nomenclature using the function miRNames, which is included in the accompanying R script.

Targetscan targets are obtained using the code below. The objects in the targetscan.Mm.eg.db package are Bimap objects, which are mappings from one set of keys (the left keys or Lkeys) to another (the right keys or Rkeys). We start by mapping the miRBase identifiers to their miRNA family names, then map the miRNA families to Entrez Gene identifiers of the targets in the TargetScan database. Several of the miRNAs of interest required slight modifications to their names prior to their mapping. The TargetScan database identifies 4,640 unique Entrez Gene identifiers as putative targets.

Mouse miRNA targets in the miRBase database are in the data frame mmTargets within the microRNA package and can be obtained using the code below. The targets are stored as Ensembl transcript identifiers. A total of 13,126 Ensembl transcripts are identified as putative targets.

Lastly, we take the intersection of the targets from TargetScan and miRBase as our set of putative targets. Ensembl transcript identifiers are firstly converted to Entrez Gene identifiers using the org.Mm.eg.db [56] Bioconductor package. The final list contains 2,080 Entrez Gene identifiers.

### Gene set analysis

As a final step in our analysis, we take the putative miRNA targets from the intersection of the TargetScan and miRBase databases and perform gene set enrichment analysis on them, using the hypergeometric test from the GOstats package [66]. Terms in the GO hierarchy are analyzed for over-representation of genes from our miRNA target list, relative to the total number from the mouse genome having that annotation. A GOHyperGParams object is created which contains the list of targets (selectedEntrezIds), the gene “universe” (entrezUniverse), the annotation database to use, the GO ontology, and direction and significance level of the test.

After the GOHyperGParams object has been created, the test can be conducted using the hyperGTest function. An html file summarizing the results can be created using the htmlReport function, which is available as Additional file 4 (“hgResult.pdf”). Particular categories of interest include GO:0060021 (palate development), GO:0048008 (platelet-derived growth factor receptor signaling pathway), GO:0060429 (epithelium development), GO:0030855 (epithelial cell differentiation), GO:0016331 (morphogenesis of embryonic epithelium), GO:0016055 (Wnt receptor signaling pathway), GO:0060828 (regulation of canonical Wnt receptor signaling pathway), GO:0008277 (regulation of G-protein coupled receptor protein signaling pathway), and GO:0007179 (transforming growth factor beta receptor signaling pathway).

As a final step, we evaluate the mature miRNA sequences and seed regions of the miRNAs which target the genes in a particular GO category. To illustrate, the GO category 0007179, transforming growth factor beta receptor signaling pathway, is used. Entrez Gene IDs belonging to this category are identified and intersected with the selected Entrez Gene IDs corresponding to cluster one of Figure 9. This results in 21 identified Entrez Gene IDs.

Next, these Entrez Gene IDs are reverse mapped back to the set of miRNAs which putatively target these genes. This produces a total of 19 identified miRNAs.

Lastly, the mature sequences and seed regions of these miRNAs are determined, using the mmSeqs database and seedRegions function in package microRNA. These sequences can be evaluated for any commonalities, to be used in determining potential targets for follow-up luciferase assays and other functional experiments [67]. In this case, the sequences are rather heterogeneous, although the seed region “GAGGUA” does show up in four of the nineteen identified miRNAs.

### Session information

It is important to note that some of the presented results may depend on the versions of the software packages that were used to produce them. The following gives the complete information of the R session under which the presented results were obtained.

## Conclusions

In this paper, we present a complete analysis of miRNA data using R and Bioconductor, including preprocessing, normalization, differential expression, clustering, identification of target genes, and gene set enrichment analysis of putative miRNA gene targets. Though there are several papers in the literature which give an overview of the analysis of miRNA data, the MmPalateMiRNA package is unique in presenting a comprehensive analysis of miRNA data which is completely reproducible. Further, while the number of packages for analyzing miRNA array data in Bioconductor is continuing to expand (see, e.g., packages LVSmiRNA [68], miRNApath [69], RmiR [70], and ExiMiR [71]), the distinguishing characteristic of this package is that it integrates many of these recent advances into one central document. Thus, this article can serve as a template for other investigators to conduct their own analysis. Important aspects of selecting a normalization algorithm for miRNA data are illustrated, along with code for producing useful diagnostic plots to select an appropriate procedure [26]. These functions are not readily accessible to users other than through the MmPalateMiRNA package. Advantages of using the limma package to fit advanced hierarchical models for testing differential expression are documented, along with code for testing comparisons between experimental groups of interest. Lastly, we illustrate the use of miRNA target databases which have been recently ported to Bioconductor for identifying putative gene targets of selected miRNAs, as well as how to test for enrichment in biological and functional categories among the putative miRNA targets. While the analysis we present here is fairly comprehensive, it is straightforward to use other software, such as Ingenuity Pathway Analysis [72] to build from the results presented in this article (see [28] as an example). The complete analysis in this article is freely available as a compendium in the form of an R package (MmPalateMiRNA, downloadable from Bioconductor [15]), along with accompanying documentation, code, and functions to perform all of the analysis.

## Availability and requirements

**Project name:** MmPalateMiRNA: An R package compendium for murine palate miRNA expression analysis**Project home page:** http://www.bioconductor.org/packages/release/bioc/html/MmPalateMiRNA.html**Operating system(s):** Platform independent**Programming language:** R**Other requirements:**R version 2.13.1 or higher [13], R packages lattice, latticeExtra, xtable, cluster, RSQLite, DBI, class, statmod, RColorBrewer, and clValid (available from CRAN [13]), and Bioconductor packages Biobase, limma, vsn, GOstats, Category, org.Mm.eg.db, microRNA, targetscan.Mm.eg.db, graph, AnnotationDbi, and multtest (available from Bioconductor [15])**License:** GNU GPL-3

## Abbreviations

CRAN: Comprehensive R Archive Network; DIANA: Divisive Analysis; GD: Gestational Day; GEO: Gene Expression Omnibus; GO: Gene Ontology; KEGG: Kyoto Encyclopedia of Genes and Genomes; miRNA: microRNA; MMU: Mus Musculus (Mouse); PAM: Partitioning Around Medoids; SAM: Significant Analysis of Microarrays; SOM: Self-Organizing Maps; SOTA: Self-Organizing Tree Algorithm; UR: Universal Reference.

## Competing interests

The authors declare that they have no competing interests.

## Authors’ contributions

GB produced the compendium MmPalateMiRNA, drafted the manuscript, and guided the statistical analysis. VP conducted the original statistical analysis. CW assisted with the biological experiments. MMP and RMG oversaw the project, and helped to draft the manuscript. PM performed the biological experiments to obtain the miRNA data, wrote the original paper on which the compendium is based, and helped draft the manuscript. All authors proofread and approved the final version of the manuscript.

## Supplementary Material

Additional file 1 **“MmPalateMiRNA_SCBM.R”.** R source code for running all of the analysis document in this manuscript. Click here for file

Additional file 2 **“ReadingTwoColorData.pdf”.** Documentation detailing how to prodcue the detail how to produce the PalateData miRNA data available in R package MmPalateMiRNA from the source data files available on GEO DataSets [31] (accession number GPL10179). Click here for file

Additional file 3 **“ReadingTwoColorData.R”.** R code to accompany Additional File 3 3, “ReadingTwoColor.pdf”. Click here for file

Additional file 4 **“hgResult.pdf”.** Significantly enriched GO biological process (BP) categories, based on the putative set of targets of differentially expressed miRNAs. P-value was based on the hypergeometric test, with all murine Entrez Gene ID entries used as the gene “universe” for comparison. For more details on how to obtain the results, see the subsection **Gene Set Analysis** under **Results and Discussion**. Click here for file
