# Supplementary material for: MmPalateMiRNA, an R package compendium illustrating analysis of miRNA microarray data
Source: Source Code Biol Med. 2013 Jan 8;8:1. doi: 10.1186/1751-0473-8-1 (PMC3654997; doi:10.1186/1751-0473-8-1)
Supplement: Additional file 2 — “ReadingTwoColorData.pdf”. Documentation detailing how to prodcue the detail how to produce the PalateData miRNA data available in R package MmPalateMiRNA from the source data files available on GEO DataSets [31] (accession number GPL10179). [file 1751-0473-8-1-S2.PDF]

Reading in Two-Color miRNA Microarray Data

Guy Brock, Partha Mukhopadhyay, Vasyl Pihur,
Cynthia Webb, Robert Greene, and Michele Pisano
University of Louisville
Louisville, KY, USA 40202

November 2, 2012

Reading in Two-Color Microarray Data

The experimental data `PalateData` is already provided and stored within the *MmPalateMiRNA* package in a compiled format, as an `RGList` object (a specialized class defined in Bioconductor package *limma* [1] for two-color microarray data). Here, we illustrate how to read in the data from the raw ImaGene files [2], available from GEO DataSets[1], accession number GPL10179. This can be accomplished several ways, but the `read.maimages` function in *limma* provides a convenient interface.

```
R> library("limma")
```

The R script in Additional File 3, "ReadingRawData.R" contains all the R code discussed in this file. The names of the raw files are read using the `list.files` function and stored as a two-column matrix, where the first column contains the names of the files containing green channel (cy3) data (the control samples) and the second column contains names of files containing red channel (cy5) data (the experimental samples). An additional feature is the possibility to incorporate spot-quality weights, via the `wt.fun` argument in the `read.maimages` function. These weights which can be used in the downstream analysis of the data, e.g. when evaluating differential expression. Here, we assign weights inversely to the "Flag" field provided for each probe, using the function `wt.function`. Note that the `rawfiles` argument specifies the directory where the raw data files are located, e.g. here "C:/palate/rawdata". The names of each data file were provided by Miltenyi

[1] GEO DataSets home, `http://www.ncbi.nlm.nih.gov/gds/`

Biotec, and correspond to experimental samples 12-1 (GD 12, replicate 1), 12-2, 12-3, 13-1, 14-3, 13-2, 13-3, 14-1, and 14-2. To avoid confusion, the samples are renamed and ordered in accordance with the experimental design.

```
R> ## Input 'x' is the raw data
R> wt.function <- function(x){
+          z <- rep(0, nrow(x))
+          z[x$Flag==0] <- 1
+          z[x$Flag==1] <- .7
+          z[x$Flag==2] <- .4
+          z[x$Flag==3] <- .2
+          z[x$Flag==4] <- .1
+          return(z)
+  }

R> ## 1. Read in the data and gene names (imagene format)
R> rawfiles <- "C:/palate/raw data"
R> imageneFiles <- matrix(list.files(rawfiles, recursive=TRUE,
+                     full.names=TRUE), byrow=TRUE, ncol=2)
R> PalateData <- read.maimages(imageneFiles, source="imagene",
+          names=c("021", "022", "023", "024", "029",
+          "033", "034", "035", "036"), wt.fun=wt.function)
R> ## Order by experiment
R> ## 12-1, 12-2, 12-3, 13-1, 13-2, 13-3, 14-1, 14-2, 14-3
R> ## 021,  022,  023,  024,  033,  034,  035,  036,  029
R> PalateData <- PalateData[,c(1:4,6:9,5)]
R> colnames(PalateData) <- c("12-1", "12-2", "12-3",
+                            "13-1", "13-2", "13-3",
+                            "14-1", "14-2", "14-3")
```

The PalateData is an RGList object, which is a list of components including the red (cy5) and green (cy3) foreground (PalateData$R and PalateData$G, respectively) and background (PalateData$Rb and Palate-Data$Gb) intensities, spot quality weights (PalateData$weights), and probe information (PalateData$genes). The raw ImaGene files have a unique identification number ("Gene ID") for each probe that is spotted on the array (this includes positive control spots and dummy, or "blank", spots). As each gene is spotted in quadruplicate on each array, a unique identifier for each probe can be created by pasting together the information on the

probe location within the chip ("Meta Row", "Meta Column", "Row", and "Column") together with the "Gene ID".

```
R> PalateData$genes$ID <- paste(PalateData$genes[,"Gene ID"],
+                              PalateData$genes[,"Meta Row"],
+                              PalateData$genes[,"Meta Column"],
+                              PalateData$genes[,"Row"],
+                              PalateData$genes[,"Column"],
+                              sep="_")
```

Probe Metadata

Information regarding the miRNA identification for each probe is contained within the corresponding PIQOR^{TM} (Parallel Identification and Quantification of RNAs) Microarray file, here "C:/palate/653.HQR" (also available from GEO, accession number GPL10179). This information can be added to the probe information for the arrays in the `PalateData` object. The file also contains information regarding the spotting structure of the array in the header of the file, which we skip over using the `skip` argument.

```
R> ## 2. read in gene names
R> fname <- "C:/palate/653.HQR"
R> piq <- read.table(fname, sep="\t", skip=31, header=TRUE,
+                    as.is=TRUE, comment.char="")
R> colnames(piq) <- c("spot.no", "ID.1", "ID.2", "Gene.Num",
+                     "Pos.X", "Pos.Y")
R> idx.match <- match(PalateData$genes$Gene, piq$Gene.Num)
R> PalateData$genes$Name <- piq$spot.no[idx.match]
R> ## create name stem for easy printing
R> tmp <- sapply(PalateData$genes$Name,
+                function(x) strsplit(x, ":"))
R> PalateData$genes$Name.stem <- sapply(tmp, function(x) x[1])
```

An additional variable, `probe.type`, was created for categorizing the probes on the arrays into different types, here "MMU miRNAs", "Other miRNAs", "Control", "Empty", and "Other". The code is suppressed here but available in Additional File 3.

```
R> table(PalateData$genes$probe.type)
```

```
MMU miRNAs Other miRNAs      Control
      2312         3000          192
     Empty        Other
       660          172
```

References

[1] G. K. Smyth. *Limma*: Linear models for microarray data. In R. Gentleman, V. Carey, S. Dudoit, R. Irizarry, and W. Huber, editors, *Bioinformatics and Computational Biology Solutions using R and Bioconductor*, pages 397–420. Springer-Verlag, New York, 2005.

[2] BioDiscovery, Inc. *ImaGene: Leading-Edge Microarray Analysis Software*. El Segundo, CA, 2011. version 9.0.
