# Supplementary material for: MmPalateMiRNA, an R package compendium illustrating analysis of miRNA microarray data
Source: Source Code Biol Med. 2013 Jan 8;8:1. doi: 10.1186/1751-0473-8-1 (PMC3654997; doi:10.1186/1751-0473-8-1)
Supplement: Additional file 4 — “hgResult.pdf”. Significantly enriched GO biological process (BP) categories, based on the putative set of targets of differentially expressed miRNAs. P-value was based on the hypergeometric test, with all murine Entrez Gene ID entries used as the gene “universe” for comparison. For more details on how to obtain the results, see the subsection Gene Set Analysis under Results and Discussion. [file 1751-0473-8-1-S4.PDF]

Gene to GO BP Conditional test for over-representation

GOBPID	Pvalue	OddsRatio	ExpCount	Count	Size	Term
GO:0044260	0.000	2.533	461	816	5512	cellular macromolecule metabolic process
GO:0034641	0.000	2.417	393	701	4674	cellular nitrogen compound metabolic process
GO:0051252	0.000	2.736	209	442	2501	regulation of RNA metabolic process
GO:0032774	0.000	2.701	217	452	2577	RNA biosynthetic process
GO:0010467	0.000	2.342	325	587	3868	gene expression
GO:0090304	0.000	2.381	303	559	3610	nucleic acid metabolic process
GO:0009987	0.000	2.493	289	483	4470	cellular process
GO:0043412	0.000	2.303	194	368	2311	macromolecule modification
GO:0009889	0.000	2.603	131	278	1661	regulation of biosynthetic process
GO:0048522	0.000	2.701	117	257	1466	positive regulation of cellular process
GO:0010558	0.000	3.033	77	190	919	negative regulation of macromolecule biosynthetic process
GO:0031327	0.000	2.974	81	195	959	negative regulation of cellular biosynthetic process
GO:0009891	0.000	2.568	106	226	1256	positive regulation of biosynthetic process
GO:0045935	0.000	2.636	97	212	1150	positive regulation of nucleobase-containing compound metabolic process
GO:0009059	0.000	2.133	192	344	2444	macromolecule biosynthetic process
GO:0045934	0.000	2.839	76	178	903	negative regulation of nucleobase-containing compound metabolic process
GO:0010604	0.000	2.719	79	180	975	positive regulation of macromolecule metabolic process
GO:0007275	0.000	2.059	182	319	2321	multicellular organismal development
GO:0031175	0.000	3.297	45	120	534	neuron projection development
GO:0010646	0.000	2.421	98	201	1175	regulation of cell communication
GO:0000122	0.000	3.163	45	117	537	negative regulation of transcription from RNA polymerase II promoter
GO:0032989	0.000	2.890	55	133	661	cellular component morphogenesis
GO:0032268	0.000	2.380	91	185	1080	regulation of cellular protein metabolic process
GO:0050789	0.000	1.693	420	582	5705	regulation of biological process
GO:0006996	0.000	2.275	102	199	1242	organelle organization
GO:0006793	0.000	2.251	101	197	1233	phosphorus metabolic process
GO:0050790	0.000	2.099	124	226	1473	regulation of catalytic activity
GO:0045944	0.000	2.583	60	133	718	positive regulation of transcription from RNA polymerase II promoter
GO:0048858	0.000	3.016	39	99	469	cell projection morphogenesis
GO:0044093	0.000	2.377	70	145	846	positive regulation of molecular function
GO:0051674	0.000	2.381	68	141	814	localization of cell
GO:0051239	0.000	2.127	94	176	1153	regulation of multicellular organismal process
GO:0019220	0.000	2.554	54	118	645	regulation of phosphate metabolic process
GO:0010627	0.000	2.696	46	105	544	regulation of intracellular protein kinase cascade

GO:0051960	0.000	2.775	41	96	485	regulation of nervous system development
GO:0006355	0.000	2.272	70	140	920	regulation of transcription, DNA-dependent
GO:0009967	0.000	2.415	59	123	699	positive regulation of signal transduction
GO:0006928	0.000	2.381	61	126	732	cellular component movement
GO:0048729	0.000	2.815	39	92	460	tissue morphogenesis
GO:0060537	0.000	3.369	25	70	303	muscle tissue development
GO:0050793	0.000	2.416	57	120	703	regulation of developmental process
GO:0001568	0.000	2.751	40	94	478	blood vessel development
GO:0008104	0.000	1.945	113	195	1342	protein localization
GO:0001701	0.000	3.397	24	66	284	in utero embryonic development
GO:0071842	0.000	2.969	31	78	411	cellular component organization at cellular level
GO:0040011	0.000	2.696	39	91	479	locomotion
GO:0044248	0.000	1.863	124	207	1488	cellular catabolic process
GO:0030036	0.000	3.117	27	70	322	actin cytoskeleton organization
GO:0061138	0.000	4.242	14	47	170	morphogenesis of a branching epithelium
GO:0000904	0.000	3.303	23	63	279	cell morphogenesis involved in differentiation
GO:0001932	0.000	2.267	60	120	717	regulation of protein phosphorylation
GO:0043067	0.000	1.965	95	167	1133	regulation of programmed cell death
GO:0045596	0.000	2.671	37	84	436	negative regulation of cell differentiation
GO:0007399	0.000	2.608	39	87	480	nervous system development
GO:0043549	0.000	2.471	45	96	532	regulation of kinase activity
GO:0051247	0.000	2.245	59	117	704	positive regulation of protein metabolic process
GO:0009894	0.000	2.408	46	97	549	regulation of catabolic process
GO:0045664	0.000	2.810	30	73	363	regulation of neuron differentiation
GO:0035556	0.000	2.177	61	118	758	intracellular signal transduction
GO:0035295	0.000	3.035	24	62	292	tube development
GO:0031401	0.000	2.334	47	97	563	positive regulation of protein modification process
GO:0030154	0.000	2.005	77	138	983	cell differentiation
GO:0046434	0.000	2.131	62	118	741	organophosphate catabolic process
GO:0007517	0.000	2.956	25	63	301	muscle organ development
GO:0034655	0.000	2.137	62	117	733	nucleobase-containing compound catabolic process
GO:0030182	0.000	2.744	30	70	363	neuron differentiation
GO:0009259	0.000	2.132	61	116	728	ribonucleotide metabolic process
GO:0009199	0.000	2.140	59	112	700	ribonucleoside triphosphate metabolic process
GO:0045893	0.000	3.031	23	58	280	positive regulation of transcription, DNA-dependent
GO:0051146	0.000	3.362	18	50	215	striated muscle cell differentiation
GO:0009144	0.000	2.125	59	112	704	purine nucleoside triphosphate metabolic process
GO:0009143	0.000	2.157	56	108	670	nucleoside triphosphate catabolic process
GO:0019538	0.000	1.617	184	269	2319	protein metabolic process
GO:0018193	0.000	2.236	49	98	589	peptidyl-amino acid modification

GO:0070727	0.000	2.076	63	116	744	cellular macromolecule localization
GO:0007519	0.000	3.700	14	43	172	skeletal muscle tissue development
GO:0007411	0.000	4.080	12	38	141	axon guidance
GO:0016568	0.000	2.683	29	67	347	chromatin modification
GO:0048754	0.000	3.782	14	41	161	branching morphogenesis of a tube
GO:0051234	0.000	1.648	157	235	1945	establishment of localization
GO:1901136	0.000	2.078	60	112	717	carbohydrate derivative catabolic process
GO:0009790	0.000	2.332	42	86	511	embryo development
GO:0080090	0.000	2.047	61	112	857	regulation of primary metabolic process
GO:0016311	0.000	2.630	29	65	340	dephosphorylation
GO:0060562	0.000	3.058	20	51	236	epithelial tube morphogenesis
GO:0030199	0.000	10.987	3	17	34	collagen fibril organization
GO:0010562	0.000	2.293	41	84	493	positive regulation of phosphorus metabolic process
GO:0060021	0.000	5.860	6	25	72	palate development
GO:0046483	0.000	1.800	95	155	1127	heterocycle metabolic process
GO:0046039	0.000	2.395	36	75	424	GTP metabolic process
GO:0006753	0.000	1.844	85	143	1017	nucleoside phosphate metabolic process
GO:0046128	0.000	2.294	39	80	469	purine ribonucleoside metabolic process
GO:0060429	0.000	2.771	24	56	284	epithelium development
GO:0072358	0.000	2.539	29	65	358	cardiovascular system development
GO:0051276	0.000	2.219	43	85	515	chromosome organization
GO:0009207	0.000	2.371	34	72	413	purine ribonucleoside triphosphate catabolic process
GO:0048523	0.000	2.369	34	71	450	negative regulation of cellular process
GO:0042327	0.000	2.230	41	81	486	positive regulation of phosphorylation
GO:0071900	0.000	2.669	25	57	294	regulation of protein serine/threonine kinase activity
GO:0009653	0.000	2.512	29	63	369	anatomical structure morphogenesis
GO:0080135	0.000	2.812	22	52	257	regulation of cellular response to stress
GO:0051129	0.000	2.658	25	57	295	negative regulation of cellular component organization
GO:0044238	0.000	1.706	114	176	1811	primary metabolic process
GO:0009154	0.000	2.343	35	72	417	purine ribonucleotide catabolic process
GO:0035637	0.000	2.111	48	90	566	multicellular organismal signaling
GO:1901069	0.000	2.323	35	72	417	guanosine-containing compound catabolic process
GO:0045860	0.000	2.585	26	58	307	positive regulation of protein kinase activity
GO:0006511	0.000	2.664	24	55	284	ubiquitin-dependent protein catabolic process
GO:0009116	0.000	2.186	42	81	494	nucleoside metabolic process
GO:0006152	0.000	2.302	35	72	420	purine nucleoside catabolic process
GO:0044281	0.000	1.564	163	234	1937	small molecule metabolic process
GO:0060548	0.000	1.997	55	99	653	negative regulation of cell death
GO:0060485	0.000	3.545	12	35	144	mesenchyme development
GO:0001655	0.000	2.909	19	46	221	urogenital system development

GO:0042454	0.000	2.269	36	72	425	ribonucleoside catabolic process
GO:1900542	0.000	2.297	34	69	403	regulation of purine nucleotide metabolic process
GO:0030811	0.000	2.529	26	57	307	regulation of nucleotide catabolic process
GO:0019219	0.000	2.670	22	51	301	regulation of nucleobase-containing compound metabolic process
GO:0042592	0.000	1.711	97	152	1152	homeostatic process
GO:0043632	0.000	2.562	25	55	293	modification-dependent macromolecule catabolic process
GO:0001503	0.000	2.611	23	53	278	ossification
GO:0023052	0.000	1.391	340	432	4173	signaling
GO:0009719	0.000	1.965	55	98	655	response to endogenous stimulus
GO:0051347	0.000	2.409	29	61	342	positive regulation of transferase activity
GO:0071822	0.000	1.888	63	109	755	protein complex subunit organization
GO:0051130	0.000	2.401	29	61	346	positive regulation of cellular component organization
GO:0001525	0.000	3.122	15	39	178	angiogenesis
GO:0010942	0.000	2.048	47	86	554	positive regulation of cell death
GO:0030855	0.000	2.637	22	50	260	epithelial cell differentiation
GO:0051248	0.000	2.277	33	66	388	negative regulation of protein metabolic process
GO:0030163	0.000	2.198	36	71	430	protein catabolic process
GO:0016337	0.000	2.483	25	54	295	cell-cell adhesion
GO:0032970	0.000	3.041	15	38	176	regulation of actin filament-based process
GO:0016202	0.000	3.965	9	27	102	regulation of striated muscle tissue development
GO:0051603	0.000	2.384	27	57	322	proteolysis involved in cellular protein catabolic process
GO:0016570	0.000	2.493	24	52	283	histone modification
GO:0033124	0.000	2.493	24	52	283	regulation of GTP catabolic process
GO:0070271	0.000	1.854	60	102	716	protein complex biogenesis
GO:0055001	0.000	3.492	10	30	125	muscle cell development
GO:0006812	0.000	1.818	64	106	757	cation transport
GO:0022402	0.000	1.806	65	108	776	cell cycle process
GO:0016477	0.000	2.245	30	60	364	cell migration
GO:0071844	0.000	2.397	24	52	300	cellular component assembly at cellular level
GO:0006357	0.000	3.286	11	31	142	regulation of transcription from RNA polymerase II promoter
GO:0009952	0.000	2.728	17	41	207	anterior/posterior pattern specification
GO:0046578	0.000	2.464	23	49	269	regulation of Ras protein signal transduction
GO:0009966	0.000	2.293	27	56	347	regulation of signal transduction
GO:0043583	0.000	2.834	16	38	186	ear development
GO:0044057	0.000	2.200	31	61	371	regulation of system process
GO:0060255	0.000	1.912	49	86	692	regulation of macromolecule metabolic process
GO:0001974	0.000	6.858	3	15	39	blood vessel remodeling
GO:0042330	0.000	2.239	29	58	345	taxis
GO:0022610	0.000	1.757	68	110	809	biological adhesion

GO:0007169	0.000	2.396	24	50	283	transmembrane receptor protein tyrosine kinase signaling pathway
GO:0032535	0.000	4.273	7	22	79	regulation of cellular component size
GO:2000112	0.000	3.033	13	33	173	regulation of cellular macromolecule biosynthetic process
GO:0060284	0.000	2.315	26	53	311	regulation of cell development
GO:0031098	0.000	2.924	14	35	167	stress-activated protein kinase signaling cascade
GO:1901135	0.000	2.286	26	53	318	carbohydrate derivative metabolic process
GO:0000226	0.000	2.456	21	46	253	microtubule cytoskeleton organization
GO:0045892	0.000	2.417	22	47	271	negative regulation of transcription, DNA-dependent
GO:0061061	0.000	3.422	10	27	116	muscle structure development
GO:0043254	0.000	2.721	16	37	187	regulation of protein complex assembly
GO:0008283	0.000	2.226	27	54	333	cell proliferation
GO:0060425	0.000	6.095	4	15	42	lung morphogenesis
GO:0033002	0.000	3.193	11	29	129	muscle cell proliferation
GO:0008285	0.000	1.997	39	70	459	negative regulation of cell proliferation
GO:0000278	0.000	1.922	44	77	522	mitotic cell cycle
GO:0070887	0.000	1.591	99	146	1174	cellular response to chemical stimulus
GO:0044085	0.000	1.862	49	84	604	cellular component biogenesis
GO:0033365	0.000	2.161	29	57	349	protein localization to organelle
GO:2000145	0.000	2.065	34	63	401	regulation of cell motility
GO:0055082	0.000	1.820	53	89	633	cellular chemical homeostasis
GO:0003015	0.000	3.211	10	28	124	heart process
GO:0007417	0.000	2.050	34	63	407	central nervous system development
GO:0048738	0.000	3.100	11	29	132	cardiac muscle tissue development
GO:0007268	0.000	1.997	37	67	439	synaptic transmission
GO:0000165	0.000	2.589	17	38	202	MAPK cascade
GO:0045597	0.000	1.924	42	73	494	positive regulation of cell differentiation
GO:0051094	0.000	2.699	15	35	181	positive regulation of developmental process
GO:0006464	0.000	2.155	28	54	358	cellular protein modification process
GO:0009888	0.000	2.317	22	46	278	tissue development
GO:0048732	0.000	2.344	22	45	257	gland development
GO:0009968	0.000	2.042	33	61	396	negative regulation of signal transduction
GO:0032870	0.000	2.310	22	46	266	cellular response to hormone stimulus
GO:0048747	0.000	3.353	9	25	107	muscle fiber development
GO:0031346	0.000	2.797	13	32	158	positive regulation of cell projection organization
GO:0014033	0.000	4.665	5	17	57	neural crest cell differentiation
GO:0043406	0.000	2.897	12	30	144	positive regulation of MAP kinase activity
GO:0048513	0.000	1.784	52	86	670	organ development
GO:0043066	0.000	1.998	34	62	409	negative regulation of apoptotic process
GO:0006338	0.000	4.296	5	18	64	chromatin remodeling
GO:0043062	0.000	2.798	13	31	153	extracellular structure organization

GO:0051169	0.000	2.209	24	48	288	nuclear transport
GO:0010033	0.000	1.493	122	171	1456	response to organic substance
GO:0022411	0.000	2.902	12	29	139	cellular component disassembly
GO:0051258	0.000	2.902	12	29	139	protein polymerization
GO:0008016	0.000	3.298	9	24	104	regulation of heart contraction
GO:0048704	0.000	3.662	7	21	84	embryonic skeletal system morphogenesis
GO:0009314	0.000	2.304	21	43	249	response to radiation
GO:0034622	0.000	2.078	29	54	343	cellular macromolecular complex assembly
GO:0051969	0.000	2.356	20	41	233	regulation of transmission of nerve impulse
GO:0007049	0.000	2.267	22	44	265	cell cycle
GO:0048864	0.000	3.257	9	24	105	stem cell development
GO:0022037	0.000	4.116	6	18	66	metencephalon development
GO:0010608	0.000	2.147	25	49	302	posttranscriptional regulation of gene expression
GO:0009791	0.000	3.217	9	24	106	post-embryonic development
GO:0048008	0.000	5.700	3	13	38	platelet-derived growth factor receptor signaling pathway
GO:0048568	0.000	3.038	10	25	117	embryonic organ development
GO:0051272	0.000	2.284	20	41	239	positive regulation of cellular component movement
GO:0046777	0.000	2.513	15	34	183	protein autophosphorylation
GO:0048736	0.000	2.751	12	29	145	appendage development
GO:0035335	0.000	3.443	7	21	88	peptidyl-tyrosine dephosphorylation
GO:0007605	0.000	3.020	10	25	116	sensory perception of sound
GO:0051091	0.000	2.684	13	30	153	positive regulation of sequence-specific DNA binding transcription factor activity
GO:0035270	0.000	3.103	9	24	109	endocrine system development
GO:0019637	0.000	1.900	36	63	442	organophosphate metabolic process
GO:0008152	0.000	1.569	87	126	1467	metabolic process
GO:0043009	0.000	3.292	8	22	98	chordate embryonic development
GO:0006606	0.000	2.456	16	35	192	protein import into nucleus
GO:0007160	0.000	3.169	9	23	103	cell-matrix adhesion
GO:0043065	0.000	1.831	41	69	488	positive regulation of apoptotic process
GO:0022414	0.000	1.581	78	116	933	reproductive process
GO:0018107	0.000	4.281	5	16	57	peptidyl-threonine phosphorylation
GO:0043467	0.000	4.569	4	15	51	regulation of generation of precursor metabolites and energy
GO:0046903	0.000	1.700	55	87	655	secretion
GO:0045445	0.000	5.277	3	13	40	myoblast differentiation
GO:0051050	0.000	1.804	43	71	507	positive regulation of transport
GO:0014031	0.000	4.819	4	14	46	mesenchymal cell development
GO:0030833	0.000	3.596	6	19	77	regulation of actin filament polymerization
GO:0061387	0.000	4.796	4	14	46	regulation of extent of cell growth
GO:0009166	0.000	1.914	34	59	406	nucleotide catabolic process

GO:0042476	0.000	3.431	7	20	84	odontogenesis
GO:0007507	0.000	2.990	9	24	114	heart development
GO:0018105	0.000	2.830	11	26	127	peptidyl-serine phosphorylation
GO:2000027	0.000	3.266	8	21	92	regulation of organ morphogenesis
GO:0051128	0.000	2.091	24	46	302	regulation of cellular component organization
GO:0035050	0.000	4.081	5	16	59	embryonic heart tube development
GO:0009953	0.000	3.248	8	21	92	dorsal/ventral pattern formation
GO:0006672	0.000	4.328	4	15	53	ceramide metabolic process
GO:0072523	0.000	2.103	24	45	286	purine-containing compound catabolic process
GO:0010906	0.000	3.658	6	18	72	regulation of glucose metabolic process
GO:0042391	0.000	2.119	23	44	273	regulation of membrane potential
GO:0001755	0.000	5.478	3	12	36	neural crest cell migration
GO:0043269	0.000	1.922	32	56	378	regulation of ion transport
GO:0048167	0.000	3.098	8	22	100	regulation of synaptic plasticity
GO:0035278	0.000	54.647	1	5	6	negative regulation of translation involved in gene silencing by miRNA
GO:0006163	0.000	1.821	39	65	468	purine nucleotide metabolic process
GO:0006974	0.000	1.812	39	66	469	response to DNA damage stimulus
GO:0035108	0.000	2.699	12	27	137	limb morphogenesis
GO:0048844	0.000	4.913	4	13	42	artery morphogenesis
GO:0021953	0.000	2.754	11	26	130	central nervous system neuron differentiation
GO:0051301	0.000	1.853	36	61	425	cell division
GO:0048638	0.000	3.745	6	17	67	regulation of developmental growth
GO:0097285	0.000	2.077	24	45	284	cell-type specific apoptotic process
GO:0001764	0.000	3.059	8	22	101	neuron migration
GO:0003206	0.000	3.059	8	22	101	cardiac chamber morphogenesis
GO:0005975	0.000	1.725	47	76	564	carbohydrate metabolic process
GO:0051241	0.000	2.003	27	49	319	negative regulation of multicellular organismal process
GO:0051493	0.000	3.266	7	20	88	regulation of cytoskeleton organization
GO:0048545	0.000	2.322	17	35	201	response to steroid hormone stimulus
GO:0048608	0.000	2.227	19	38	226	reproductive structure development
GO:0007409	0.000	3.242	7	20	89	axonogenesis
GO:0043506	0.000	3.900	5	16	61	regulation of JUN kinase activity
GO:0010002	0.000	12.761	1	7	13	cardioblast differentiation
GO:0031324	0.000	2.209	19	38	241	negative regulation of cellular metabolic process
GO:0090183	0.000	4.385	4	14	49	regulation of kidney development
GO:0003002	0.000	5.150	3	12	38	regionalization
GO:0048856	0.000	1.859	34	58	461	anatomical structure development
GO:0021587	0.000	5.737	3	11	32	cerebellum morphogenesis
GO:0001890	0.000	2.904	9	23	110	placenta development

GO:0003007	0.000	4.051	5	15	56	heart morphogenesis
GO:0030832	0.000	3.182	7	20	89	regulation of actin filament length
GO:0060711	0.000	5.056	3	12	38	labyrinthine layer development
GO:0032874	0.000	4.011	5	15	56	positive regulation of stress-activated MAPK cascade
GO:0006897	0.000	1.869	33	56	387	endocytosis
GO:0006397	0.000	1.986	26	47	308	mRNA processing
GO:0048534	0.000	4.940	3	12	39	hemopoietic or lymphoid organ development
GO:0021680	0.000	8.753	2	8	18	cerebellar Purkinje cell layer development
GO:0050773	0.000	3.518	6	17	70	regulation of dendrite development
GO:0008219	0.000	1.832	34	58	422	cell death
GO:0050768	0.000	3.208	7	19	84	negative regulation of neurogenesis
GO:0021846	0.000	6.082	2	10	28	cell proliferation in forebrain
GO:0017148	0.000	4.147	4	14	51	negative regulation of translation
GO:0035272	0.000	4.451	4	13	45	exocrine system development
GO:0045667	0.000	2.876	9	22	106	regulation of osteoblast differentiation
GO:0008284	0.000	1.712	45	71	534	positive regulation of cell proliferation
GO:0040017	0.000	2.116	20	39	242	positive regulation of locomotion
GO:0010921	0.000	3.049	8	20	92	regulation of phosphatase activity
GO:0030155	0.000	2.088	21	40	251	regulation of cell adhesion
GO:0016055	0.000	2.240	17	34	202	Wnt receptor signaling pathway
GO:0016310	0.000	2.078	21	40	264	phosphorylation
GO:0044087	0.000	2.927	8	21	101	regulation of cellular component biogenesis
GO:0060322	0.000	4.038	4	14	52	head development
GO:0032879	0.000	1.964	26	46	315	regulation of localization
GO:0001947	0.000	4.695	3	12	40	heart looping
GO:0008154	0.000	2.918	8	21	100	actin polymerization or depolymerization
GO:0006886	0.000	1.876	30	52	360	intracellular protein transport
GO:0030335	0.000	2.144	19	37	227	positive regulation of cell migration
GO:0045974	0.000	27.322	1	5	7	regulation of translation, ncRNA-mediated
GO:0051494	0.000	3.237	7	18	79	negative regulation of cytoskeleton organization
GO:0030324	0.000	2.901	8	21	101	lung development
GO:0006916	0.000	2.189	18	35	211	anti-apoptosis
GO:0060479	0.000	7.957	2	8	19	lung cell differentiation
GO:2000826	0.000	7.957	2	8	19	regulation of heart morphogenesis
GO:0060828	0.000	2.663	10	24	123	regulation of canonical Wnt receptor signaling pathway
GO:0007389	0.000	3.093	7	19	88	pattern specification process
GO:0051216	0.000	2.983	8	20	94	cartilage development
GO:0030097	0.000	1.698	44	70	526	hemopoiesis
GO:0042474	0.000	6.566	2	9	24	middle ear morphogenesis
GO:0032869	0.000	2.882	8	21	101	cellular response to insulin stimulus

GO:0030010	0.000	3.509	6	16	66	establishment of cell polarity
GO:0055080	0.000	1.813	33	56	397	cation homeostasis
GO:0048813	0.000	3.329	6	17	73	dendrite morphogenesis
GO:0007009	0.000	3.066	7	19	87	plasma membrane organization
GO:0034329	0.000	3.066	7	19	87	cell junction assembly
GO:0014902	0.000	4.533	3	12	41	myotube differentiation
GO:0007267	0.000	2.768	9	22	111	cell-cell signaling
GO:0007265	0.000	3.466	6	16	67	Ras protein signal transduction
GO:0048646	0.000	2.217	16	33	203	anatomical structure formation involved in morphogenesis
GO:0016043	0.000	1.939	25	45	347	cellular component organization
GO:0048636	0.000	9.570	1	7	15	positive regulation of muscle organ development
GO:0051147	0.000	2.927	8	20	95	regulation of muscle cell differentiation
GO:0034623	0.000	3.021	7	19	88	cellular macromolecular complex disassembly
GO:0045995	0.000	3.271	6	17	74	regulation of embryonic development
GO:0007216	0.000	13.119	1	6	11	G-protein coupled glutamate receptor signaling pathway
GO:0010560	0.000	13.119	1	6	11	positive regulation of glycoprotein biosynthetic process
GO:0045815	0.000	13.119	1	6	11	positive regulation of gene expression, epigenetic
GO:0007611	0.000	2.344	14	29	165	learning or memory
GO:0030902	0.000	4.858	3	11	36	hindbrain development
GO:0035051	0.000	3.591	5	15	61	cardiac cell differentiation
GO:0043491	0.000	4.403	4	12	42	protein kinase B signaling cascade
GO:0031032	0.000	4.069	4	13	48	actomyosin structure organization
GO:0000084	0.000	4.818	3	11	36	S phase of mitotic cell cycle
GO:0048675	0.000	3.574	5	15	61	axon extension
GO:0031331	0.000	2.888	8	20	96	positive regulation of cellular catabolic process
GO:0034762	0.000	1.822	31	53	374	regulation of transmembrane transport
GO:0007632	0.000	4.381	4	12	42	visual behavior
GO:0003001	0.000	1.906	26	46	312	generation of a signal involved in cell-cell signaling
GO:0006814	0.000	2.243	15	31	183	sodium ion transport
GO:0060341	0.000	1.630	50	76	592	regulation of cellular localization
GO:0008277	0.000	2.850	8	20	97	regulation of G-protein coupled receptor protein signaling pathway
GO:0050770	0.000	2.850	8	20	97	regulation of axonogenesis
GO:0006813	0.000	2.349	13	28	159	potassium ion transport
GO:0018108	0.000	2.137	18	34	209	peptidyl-tyrosine phosphorylation
GO:0032319	0.000	3.037	7	18	83	regulation of Rho GTPase activity
GO:0016032	0.000	2.534	11	24	128	viral reproduction
GO:0007368	0.000	3.160	6	17	76	determination of left/right symmetry
GO:0007369	0.000	2.474	11	25	136	gastrulation
GO:0002053	0.000	4.633	3	11	37	positive regulation of mesenchymal cell proliferation

GO:0051049	0.000	2.370	13	27	156	regulation of transport
GO:0016192	0.000	1.749	36	58	429	vesicle-mediated transport
GO:0019233	0.000	2.814	8	20	98	sensory perception of pain
GO:0030900	0.000	2.072	19	36	228	forebrain development
GO:0021695	0.000	8.536	1	7	16	cerebellar cortex development
GO:0010769	0.000	3.127	6	17	77	regulation of cell morphogenesis involved in differentiation
GO:0021697	0.000	8.506	1	7	16	cerebellar cortex formation
GO:0070875	0.000	8.506	1	7	16	positive regulation of glycogen metabolic process
GO:0048260	0.000	5.793	2	9	26	positive regulation of receptor-mediated endocytosis
GO:0065008	0.000	1.578	56	83	702	regulation of biological quality
GO:0051345	0.000	2.226	15	30	180	positive regulation of hydrolase activity
GO:0043393	0.000	2.778	8	20	99	regulation of protein binding
GO:0051649	0.000	1.658	44	68	535	establishment of localization in cell
GO:0048752	0.000	18.214	1	5	8	semicircular canal morphogenesis
GO:0035148	0.000	2.551	10	23	122	tube formation
GO:0050808	0.000	2.551	10	23	122	synapse organization
GO:0006915	0.000	1.854	27	47	338	apoptotic process
GO:1901214	0.000	2.336	13	27	154	regulation of neuron death
GO:0043392	0.000	4.976	3	10	32	negative regulation of DNA binding
GO:0001822	0.000	3.079	7	17	78	kidney development
GO:0048742	0.000	4.107	4	12	44	regulation of skeletal muscle fiber development
GO:0045786	0.000	1.927	24	42	282	negative regulation of cell cycle
GO:0002376	0.000	1.404	109	145	1295	immune system process
GO:0001667	0.000	3.071	7	17	78	ameboidal cell migration
GO:0043410	0.000	2.477	11	24	131	positive regulation of MAPK cascade
GO:0060911	0.000	10.932	1	6	12	cardiac cell fate commitment
GO:0071108	0.000	10.932	1	6	12	protein K48-linked deubiquitination
GO:0006333	0.000	2.946	7	18	85	chromatin assembly or disassembly
GO:0009799	0.000	2.946	7	18	85	specification of symmetry
GO:0031400	0.000	1.978	22	39	256	negative regulation of protein modification process
GO:0070372	0.000	2.597	10	22	115	regulation of ERK1 and ERK2 cascade
GO:0007269	0.000	3.056	7	17	78	neurotransmitter secretion
GO:0065004	0.000	3.056	7	17	78	protein-DNA complex assembly
GO:0006650	0.000	2.317	13	27	155	glycerophospholipid metabolic process
GO:0044255	0.000	1.565	56	83	670	cellular lipid metabolic process
GO:0060297	0.000	43.696	0	4	5	regulation of sarcomere organization
GO:0001706	0.000	5.471	2	9	27	endoderm formation
GO:0019080	0.000	5.471	2	9	27	viral genome expression
GO:0048565	0.000	2.903	7	18	86	digestive tract development
GO:0051897	0.000	3.486	5	14	58	positive regulation of protein kinase B signaling cascade

GO:0032502	0.000	2.535	10	22	136	developmental process
GO:0021915	0.000	2.387	12	25	140	neural tube development
GO:0003151	0.000	3.982	4	12	45	outflow tract morphogenesis
GO:0035019	0.000	3.982	4	12	45	somatic stem cell maintenance
GO:0000271	0.000	3.006	7	17	79	polysaccharide biosynthetic process
GO:0070838	0.000	1.871	25	44	303	divalent metal ion transport
GO:0030330	0.000	3.288	5	15	65	DNA damage response, signal transduction by p53 class mediator
GO:0043434	0.000	2.213	15	29	173	response to peptide hormone stimulus
GO:0032881	0.000	4.759	3	10	33	regulation of polysaccharide metabolic process
GO:0033627	0.000	4.301	3	11	39	cell adhesion mediated by integrin
GO:0042472	0.000	2.868	7	18	87	inner ear morphogenesis
GO:0009101	0.000	2.235	14	28	166	glycoprotein biosynthetic process
GO:0090066	0.000	2.528	10	22	119	regulation of anatomical structure size
GO:0003012	0.000	2.130	16	31	191	muscle system process
GO:0001657	0.000	2.959	7	17	80	ureteric bud development
GO:0043484	0.000	3.409	5	14	59	regulation of RNA splicing
GO:0051259	0.000	1.778	30	50	360	protein oligomerization
GO:0033143	0.000	3.865	4	12	46	regulation of intracellular steroid hormone receptor signaling pathway
GO:0051384	0.000	3.865	4	12	46	response to glucocorticoid stimulus
GO:0051325	0.000	2.116	16	31	192	interphase
GO:0046907	0.000	2.004	19	35	231	intracellular transport
GO:0006875	0.000	1.857	25	43	298	cellular metal ion homeostasis
GO:0016331	0.000	2.326	12	25	143	morphogenesis of embryonic epithelium
GO:0035264	0.000	2.374	11	24	135	multicellular organism growth
GO:0021533	0.000	9.388	1	6	13	cell differentiation in hindbrain
GO:0048663	0.000	3.334	5	14	60	neuron fate commitment
GO:0015031	0.000	1.633	42	65	513	protein transport
GO:0001824	0.000	3.161	6	15	67	blastocyst development
GO:0070201	0.000	1.864	24	42	290	regulation of establishment of protein localization
GO:0043123	0.000	2.612	9	20	104	positive regulation of I-kappaB kinase/NF-kappaB cascade
GO:0071214	0.000	2.612	9	20	104	cellular response to abiotic stimulus
GO:0046627	0.000	5.834	2	8	23	negative regulation of insulin receptor signaling pathway
GO:0042733	0.000	3.754	4	12	47	embryonic digit morphogenesis
GO:0046822	0.000	2.352	11	24	136	regulation of nucleocytoplasmic transport
GO:0007379	0.000	13.660	1	5	9	segment specification
GO:0060052	0.000	13.660	1	5	9	neurofilament cytoskeleton organization
GO:0060693	0.000	13.660	1	5	9	regulation of branching involved in salivary gland morphogenesis

GO:0001666	0.000	2.404	11	23	128	response to hypoxia
GO:0045637	0.000	2.404	11	23	128	regulation of myeloid cell differentiation
GO:0016458	0.000	2.672	8	19	97	gene silencing
GO:0001953	0.000	6.959	2	7	18	negative regulation of cell-matrix adhesion
GO:0007626	0.000	2.213	14	27	161	locomotory behavior
GO:0031344	0.000	2.584	9	20	106	regulation of cell projection organization
GO:0070646	0.000	2.972	6	16	75	protein modification by small protein removal
GO:0065003	0.000	1.679	36	57	439	macromolecular complex assembly
GO:0007179	0.000	2.504	9	21	113	transforming growth factor beta receptor signaling pathway
GO:0051495	0.000	2.741	8	18	90	positive regulation of cytoskeleton organization
GO:0010001	0.000	2.331	12	24	137	glial cell differentiation
GO:0046847	0.000	3.650	4	12	48	filopodium assembly
GO:0008015	0.000	2.086	16	30	189	blood circulation
GO:0043280	0.001	2.704	8	18	91	positive regulation of cysteine-type endopeptidase activity involved in apoptotic process
GO:0003279	0.001	3.390	5	13	55	cardiac septum development
GO:0043255	0.001	3.390	5	13	55	regulation of carbohydrate biosynthetic process
GO:0048661	0.001	3.390	5	13	55	positive regulation of smooth muscle cell proliferation
GO:0060491	0.001	3.390	5	13	55	regulation of cell projection assembly
GO:0034728	0.001	3.044	6	15	69	nucleosome organization
GO:0048255	0.001	5.469	2	8	24	mRNA stabilization
GO:0048745	0.001	5.469	2	8	24	smooth muscle tissue development
GO:0045936	0.001	1.993	18	33	215	negative regulation of phosphate metabolic process
GO:0030308	0.001	2.341	11	23	131	negative regulation of cell growth
GO:0043112	0.001	2.521	9	20	107	receptor metabolic process
GO:2000116	0.001	2.164	14	27	164	regulation of cysteine-type endopeptidase activity
GO:0008654	0.001	2.390	10	22	123	phospholipid biosynthetic process
GO:0007243	0.001	2.666	8	18	95	intracellular protein kinase cascade
GO:0003156	0.001	4.209	3	10	36	regulation of organ formation
GO:0010676	0.001	4.209	3	10	36	positive regulation of cellular carbohydrate metabolic process
GO:0045765	0.001	2.196	13	26	156	regulation of angiogenesis
GO:0006275	0.001	2.667	8	18	92	regulation of DNA replication
GO:0048013	0.001	4.689	3	9	30	ephrin receptor signaling pathway
GO:0060325	0.001	4.689	3	9	30	face morphogenesis
GO:0060428	0.001	4.689	3	9	30	lung epithelium development
GO:0048486	0.001	8.198	1	6	14	parasympathetic nervous system development
GO:0060037	0.001	8.198	1	6	14	pharyngeal system development
GO:0040008	0.001	1.729	30	49	365	regulation of growth
GO:0006468	0.001	1.814	25	42	308	protein phosphorylation

GO:0030422	0.001	Inf	0	3	3	production of siRNA involved in RNA interference
GO:0030423	0.001	Inf	0	3	3	targeting of mRNA for destruction involved in RNA interference
GO:0048671	0.001	Inf	0	3	3	negative regulation of collateral sprouting
GO:0071526	0.001	6.379	2	7	19	semaphorin-plexin signaling pathway
GO:0007254	0.001	2.433	10	21	116	JNK cascade
GO:0031323	0.001	2.067	15	29	222	regulation of cellular metabolic process
GO:0043588	0.001	3.311	5	13	56	skin development
GO:0070647	0.001	1.715	31	50	373	protein modification by small protein conjugation or removal
GO:0048562	0.001	2.368	10	22	125	embryonic organ morphogenesis
GO:0001942	0.001	2.740	7	17	85	hair follicle development
GO:0032320	0.001	2.366	10	22	124	positive regulation of Ras GTPase activity
GO:0033554	0.001	1.904	20	36	250	cellular response to stress
GO:0021707	0.001	21.847	1	4	6	cerebellar granule cell differentiation
GO:0048251	0.001	21.847	1	4	6	elastic fiber assembly
GO:0051450	0.001	21.847	1	4	6	myoblast proliferation
GO:0060510	0.001	21.847	1	4	6	Type II pneumocyte differentiation
GO:0034220	0.001	1.512	57	81	673	ion transmembrane transport
GO:0042633	0.001	2.631	8	18	93	hair cycle
GO:0048639	0.001	3.763	4	11	43	positive regulation of developmental growth
GO:0016049	0.001	2.039	16	30	193	cell growth
GO:0022604	0.001	2.541	8	19	102	regulation of cell morphogenesis
GO:0048705	0.001	2.529	9	19	102	skeletal system morphogenesis
GO:0022404	0.001	2.701	7	17	86	molting cycle process
GO:0007257	0.001	5.147	2	8	25	activation of JUN kinase activity
GO:0050881	0.001	5.147	2	8	25	musculoskeletal movement
GO:0072006	0.001	3.067	5	14	64	nephron development
GO:0021675	0.001	3.236	5	13	57	nerve development
GO:0051716	0.001	1.229	294	343	3690	cellular response to stimulus
GO:0031061	0.001	10.927	1	5	10	negative regulation of histone methylation
GO:0035024	0.001	10.927	1	5	10	negative regulation of Rho protein signal transduction
GO:0048643	0.001	10.927	1	5	10	positive regulation of skeletal muscle tissue development
GO:0051386	0.001	10.927	1	5	10	regulation of nerve growth factor receptor signaling pathway
GO:0051531	0.001	10.927	1	5	10	NFAT protein import into nucleus
GO:0060413	0.001	10.927	1	5	10	atrial septum morphogenesis
GO:0060536	0.001	10.927	1	5	10	cartilage morphogenesis
GO:0007435	0.001	4.476	3	9	31	salivary gland morphogenesis
GO:0055013	0.001	4.476	3	9	31	cardiac muscle cell development
GO:0033044	0.001	2.596	8	18	94	regulation of chromosome organization

GO:0051051	0.001	1.804	24	41	291	negative regulation of transport
GO:0010927	0.001	2.321	11	22	126	cellular component assembly involved in morphogenesis
GO:0016044	0.001	1.942	18	33	221	cellular membrane organization
GO:0042060	0.001	1.939	18	33	220	wound healing
GO:0050678	0.001	1.980	17	31	203	regulation of epithelial cell proliferation
GO:0048557	0.001	5.888	2	7	20	embryonic digestive tract morphogenesis
GO:0030522	0.001	2.253	11	23	135	intracellular receptor mediated signaling pathway
GO:0006941	0.001	3.007	5	14	65	striated muscle contraction
GO:0003231	0.001	2.563	8	18	95	cardiac ventricle development
GO:0007498	0.001	2.563	8	18	95	mesoderm development
GO:0030316	0.001	3.164	5	13	58	osteoclast differentiation
GO:0046545	0.001	2.480	9	19	103	development of primary female sexual characteristics
GO:0019228	0.001	2.350	10	21	119	regulation of action potential in neuron
GO:0051100	0.001	3.924	3	10	38	negative regulation of binding
GO:0051924	0.001	2.161	13	25	152	regulation of calcium ion transport
GO:0006200	0.001	1.867	21	36	248	ATP catabolic process
GO:0048699	0.001	3.138	5	13	61	generation of neurons
GO:0035304	0.001	7.287	1	6	15	regulation of protein dephosphorylation
GO:0055085	0.001	1.924	19	33	225	transmembrane transport
GO:0043487	0.001	3.908	3	10	38	regulation of RNA stability
GO:0060993	0.001	3.908	3	10	38	kidney morphogenesis
GO:0043547	0.001	2.342	10	21	120	positive regulation of GTPase activity
GO:0033157	0.001	2.195	12	24	144	regulation of intracellular protein transport
GO:0000279	0.001	1.619	37	57	445	M phase
GO:0001662	0.001	4.861	2	8	26	behavioral fear response
GO:0046782	0.001	4.861	2	8	26	regulation of viral transcription
GO:0032872	0.001	2.713	7	16	81	regulation of stress-activated MAPK cascade
GO:0051058	0.001	4.281	3	9	32	negative regulation of small GTPase mediated signal transduction
GO:0010950	0.001	2.530	8	18	96	positive regulation of endopeptidase activity
GO:0050880	0.001	2.327	10	21	120	regulation of blood vessel size
GO:0030178	0.001	2.451	9	19	104	negative regulation of Wnt receptor signaling pathway
GO:0035113	0.001	2.384	9	20	112	embryonic appendage morphogenesis
GO:0045787	0.001	2.384	9	20	112	positive regulation of cell cycle
GO:0000186	0.001	3.541	4	11	45	activation of MAPKK activity
GO:0043525	0.001	3.541	4	11	45	positive regulation of neuron apoptotic process
GO:0061035	0.001	3.541	4	11	45	regulation of cartilage development
GO:0016567	0.001	1.702	29	47	351	protein ubiquitination
GO:0001837	0.001	2.949	6	14	66	epithelial to mesenchymal transition
GO:0046620	0.001	2.949	6	14	66	regulation of organ growth
